# Post-Exercise Heart Rate Recovery Independently Predicts Clinical Outcome in Patients with Acute Decompensated Heart Failure

**DOI:** 10.1371/journal.pone.0154534

**Published:** 2016-05-02

**Authors:** Jong-Chan Youn, Hye Sun Lee, Suk-Won Choi, Seong-Woo Han, Kyu-Hyung Ryu, Eui-Cheol Shin, Seok-Min Kang

**Affiliations:** 1 Division of Cardiology, Dongtan Sacred Heart Hospital, Hallym University College of Medicine, Hwaseong, Republic of Korea; 2 Division of Cardiology, Severance Cardiovascular Hospital, Yonsei University College of Medicine, Seoul, Republic of Korea; 3 Department of Biostatistics, Yonsei University College of Medicine, Seoul, Republic of Korea; 4 Laboratory of Immunology and Infectious Diseases, Graduate School of Medical Science and Engineering, KAIST, Daejeon, Republic of Korea; Sapienza University of Rome, ITALY

## Abstract

**Background:**

Post-exercise heart rate recovery (HRR) is an index of parasympathetic function associated with clinical outcome in patients with chronic heart failure. However, its relationship with the pro-inflammatory response and prognostic value in consecutive patients with acute decompensated heart failure (ADHF) has not been investigated.

**Methods:**

We measured HRR and pro-inflammatory markers in 107 prospectively and consecutively enrolled, recovered ADHF patients (71 male, 59 ± 15 years, mean ejection fraction 28.9 ± 14.2%) during the pre-discharge period. The primary endpoint included cardiovascular (CV) events defined as CV mortality, cardiac transplantation, or rehospitalization due to HF aggravation.

**Results:**

The CV events occurred in 30 (28.0%) patients (5 cardiovascular deaths and 7 cardiac transplantations) during the follow-up period (median 214 days, 11–812 days). When the patients with ADHF were grouped by HRR according to the Contal and O’Quigley’s method, low HRR was shown to be associated with significantly higher levels of serum monokine-induced by gamma interferon (MIG) and poor clinical outcome. Multivariate Cox regression analysis revealed that low HRR was an independent predictor of CV events in both enter method and stepwise method. The addition of HRR to a model significantly increased predictability for CV events across the entire follow-up period.

**Conclusion:**

Impaired post-exercise HRR is associated with a pro-inflammatory response and independently predicts clinical outcome in patients with ADHF. These findings may explain the relationship between autonomic dysfunction and clinical outcome in terms of the inflammatory response in these patients.

## Introduction

Autonomic imbalance in heart failure is characterized by increased sympathetic activity and withdrawal of parasympathetic activity. This autonomic imbalance is associated with progression of heart failure and a worse clinical outcome [1]. While beta-blockers have found a place as a leading disease-modifying therapy that impacts the sympathetic nervous system, far less is known about the withdrawal of parasympathetic activity. Moreover, it is difficult to measure parasympathetic function directly. Parasympathetic activity can be measured crudely by examining responses to vagal nerve stimulation or pharmacologic blockade, heart rate variability, and post-exercise heart rate recovery (HRR) [2].

Among these, post-exercise HRR provides a non-invasive and clinically feasible method to quantitatively assess parasympathetic function. Impaired heart rate deceleration after exercise cessation is associated with poor clinical outcome in subjects referred for exercise testing regardless of cardiovascular disease history [3–5] or in patients with chronic heart failure [6–11]. However, its prognostic value has not been confirmed in prospectively and consecutively enrolled acute decompensated heart failure (ADHF) patients. Moreover, the underlying mechanism of how impaired HRR is associated with poor clinical outcome is not well understood. One contributing factor could be that impaired HRR appears to correlate with an excessive pro-inflammatory status. According to the recent concept of ‘cholinergic anti-inflammatory pathway’, immunity is coordinated by neural circuits that operate reflexively, and this well-established neural circuit terminates excessive pro-inflammatory cytokine responses, preventing immune-mediated damage [12,13]. Therefore, decreased parasympathetic activity may result in pro-inflammatory responses and increased morbidity and mortality [14–16]. Thus, we evaluated the prognostic value of post-exercise HRR, which is an index of parasympathetic function, in relation with the pro-inflammatory response in prospectively and consecutively enrolled, recovered ADHF patients.

## Methods

### Study population

Patients who were diagnosed with ADHF at Severance Cardiovascular Hospital were prospectively and consecutively enrolled between May 2012 and April 2014. Patients who displayed rapid onset of signs or symptoms of heart failure and one of the following criteria were eligible for the study: (i) lung congestion or (ii) objective findings of left ventricular systolic dysfunction or structural heart disease. Lung congestion was defined as ‘congestion’ on a chest X-ray or as rales on physical examination. Recovered ambulatory ADHF patients during hospitalization were eligible for the study. Patients who could not perform the cardiopulmonary exercise test (CPET) were excluded from this study. The primary endpoint included cardiovascular (CV) events defined as a composite of death, rehospitalization due to worsening heart failure, or urgent cardiac transplantation.

### Ethics statement

The study protocol was approved by the institutional review board (IRB) of Yonsei University College of Medicine, Severance Hospital, Seoul, Republic of Korea and IRB number was 4-2012-0027. All subjects provided written informed consent to participate in this study.

### Biochemical and echocardiographic analysis

Blood samples were obtained after an 8-h overnight fast by venipuncture into plain and EDTA tubes. Complete blood count, serum sodium, total cholesterol, albumin and serum creatinine (Cr) were measured using standard automated laboratory techniques. The blood samples were tested with NT-proBNP, which was kept at 4°C, using an electrochemiluminescence immunoassay (Elecsys proBNP; Roche Diagnostics, Basel, Switzerland); intra- and inter-assay coefficients of variations (CVs) were <1.3% and <1.7%, respectively. Plasma high sensitivity C-reactive protein (hsCRP) levels were measured using an immunonephelometric method on a BNII analyzer (Dade-Behring, Germany) with the manufacturer’s reagents. The detection limit for hsCRP was 0.2 mg/L; intra- and inter-assay CVs were <5%, respectively.

Almost all echocardiographic measurements were performed at a relatively consistent time point for the entire cohort, that is, on the day of admission or the day after admission. Left ventricular ejection fraction (LVEF) was measured using the modified Quinones method. In patients with regional wall motion abnormalities, the LVEF was calculated using Simpson’s biplane method with apical four- and two-chamber views.

### Cytometric bead array

We tried to assess the relationship between levels of pro-inflammatory markers and clinical outcome in relation with HRR in this study. Blood for measuring various pro-inflammatory chemokines was drawn on the day of or before discharge, at about the same time as the CPET measurements. The concentrations of various serum chemokine markers (monokine-induced by gamma interferon [MIG] (intra- and inter-assay CVs; 8.7% and 5.2%, respectively), interferon gamma-induced protein 10 [IP-10] (intra- and inter-assay CVs; 4.0% and 4.0%, respectively), chemokine (C-X3-C motif) ligand 1 [CX3CL1] (intra- and inter-assay CVs; 2.0% and 3.0%, respectively), monocyte chemotactic protein 1 [MCP1] (intra- and inter-assay CVs; 4.0% and 6.9%, respectively), macrophage inflammatory protein 1α [MIP1α] (intra- and inter-assay CVs; 4.0% and 4.0%, respectively), macrophage inflammatory protein 1β [MIP1β] (intra- and inter-assay CVs; 3.0% and 3.0%, respectively), regulated on activation normal T cell expressed and secreted [RANTES] (intra- and inter-assay CVs; 6.0% and 4.2%, respectively)), granzyme B (intra- and inter-assay CVs; 2.0% and 5.0%, respectively), and tumour necrosis factor-α (TNF-α) (intra- and inter-assay CVs; 6.0% and 8.0%, respectively) were measured by flow cytometry using the BD cytometric bead array technique. Sample processing was performed according to the manufacturer-supplied instructions (BD Biosciences, San Jose, CA). Briefly, 50 μl of mixed capture beads and 50 μl of each serum sample were incubated for 1 h at room temperature (RT). Next, 50 μl of mixed phycoerythrin detection reagents were added to the bead-sample mixture and incubated for 2 hour at RT. The samples were washed and assessed with an LSR II Flow Cytometer (BD Biosciences, San Jose, CA). The data were analysed with FlowJo software version 9.2 for Mac (TreeStar, Ashland, OR).

### Assessment of post-exercise heart rate recovery (HRR)

A symptom-limited CPET was performed on a treadmill according to the modified Bruce ramp protocol. The CPET was performed after “clinical stabilization” according to current medical guidelines. Clinical stabilization was defined as readiness for discharge from the hospital. Congestion was absent, and a stable, oral diuretic regimen was established for at least 48 hours. The CPET was performed at a relatively consistent time point, that is, on the day of or before discharge. Patients were strongly encouraged to achieve a peak respiratory exchange ratio (RER) > 1.10. Expired gases were collected continuously throughout exercise and analysed for ventilator volume, oxygen (O_2_) content, and carbon dioxide (CO_2_) content using a calibrated metabolic cart (Quark CPET, COSMED, Chicago, IL, USA). Expired gases were reported every 15 seconds. During the exercise test, monitoring consisted of continuous 12-lead electrocardiography, manual blood pressure measurements and heart rate recordings at every stage. CPET was terminated according to the following criteria: patient request, ventricular tachycardia, ≥2 mm of horizontal or down-sloping ST segment depression, or a drop in systolic blood pressure ≥20 mm/Hg during exercise. A qualified exercise physiologist conducted each test with the supervision of a physician. The following variables were derived from the CPET results: peak oxygen consumption (peak VO_2_); peak RER, defined by the ratio of CO_2_ production to O_2_ consumption at peak effort; VE/VCO_2_ slope, defined as the slope of the increase in peak ventilation/increase in CO_2_ production throughout exercise. After completion of exercise, the treadmill was stopped within 20 seconds and all subjects recovered in a seated position. HRR was calculated as the difference between heart rate at peak exercise and after 1 or 2 minutes of recovery after CPET. Because there have been no reports regarding HRR in ADHF patients, we analysed both HRR(1 min) and HRR(2 min) in this study.

### Statistical analysis

Continuous variables are summarized as the mean ± standard deviation. Categorical variables are summarized as a percentage of the total group. Discrete variables were compared using the chi-squared method. Regarding the cut-off value of the post-exercise HRR, we chose a cut-off point according to the Contal and O’Quigley's method [17] to maximize the hazard ratio (HRR[1 min] < 13, HRR[2 min] < 27). The cumulative incidence of CV events was assessed with the Kaplan-Meier method. The statistical significance of the curves was calculated using the log-rank test. Univariate and multivariate Cox regression analysis were performed to identify independent predictors of CV events in patients with ADHF. Because HRR(1min) and HRR(2min) do not show normal distribution even after logarithmic transformation, we analysed HRR(1min) and HRR(2min) as a categorical variables in Cox regression analysis. In enter method multivariate Cox regression analysis, all significant variables in the univariate Cox analysis were included to adjust relevant variables as many as possible. In addition, stepwise method multivariate Cox regression analyses were also performed to evaluate the independent associations of HRR(1min) and HRR(2min) with the most relevant minimum variables to avoid the over-fitting. We compared a model with HRR (full model) to a model without HRR (reduced model) using the likelihood ratio test. To further evaluate the predictability of HRR across the entire follow-up period, we applied a time-dependent ROC curve method for censored CV events data. We then compared the global concordance probability (integrated area under the curve, iAUC) of the model with HRR(1 min) and HRR(2 min) to that of the model without HRR. iAUC is a weighted average of the area under the curve (AUC) across a period of follow-up that measures the predictive accuracy of a model during that period. The differences and 95% confidence interval were calculated by bootstrapping method. Two-sided p values of less than 0.05 and excluding 0 in the 95% confidence interval were considered to indicate statistical significance. All statistical analyses were performed with SPSS version 20.0 (IBM Corporation, Armonk, NY, USA), SAS (version 9.2, SAS Inc., Cary, NC, USA), and the R Statistical Package (Institute for Statistics and Mathematics, Vienna, Austria, ver. 3.1.2, www.R-project.org).

## Results

### Baseline characteristics of study subjects according to CV events

The CV events occurred in 30 (28.0%) patients (5 cardiovascular deaths and 7 cardiac transplantations) during the follow-up period (median 214 days, 11–812 days). Clinical characteristics, echocardiographic, laboratory, and cardiopulmonary exercise test parameters of the 107 ADHF patients studied (71 male, 59 ± 15 years, mean ejection fraction 28.9 ± 14.2%) are summarized in Table 1 according to CV events. Patients with CV events were more likely to exhibit exacerbated chronic HF rather than de novo HF, showed significantly lower BMI, hemoglobin level, and higher NT-proBNP. The beta-blocker prescription rate of patients with CV events was lower than that of patients without CV events. CPET parameters, including HRR(1 min) and HRR(2 min), differed significantly between the two groups. Various markers for the pro-inflammatory response, including MIG, IP-10, CXCL1, MCP-1, MIP1α, MIP1β, RANTES, granzyme B, and TNF-α, showed no significant differences between patients with and without CV events.

**Table 1:** Clinical characteristics, echocardiographic, laboratory and cardiopulmonary exercise test parameters according to CV events in study subjects.

	CV events (N = 30, 28.0%)	No CV event (N = 77, 72.0%)	P-value
Age (years)	62 ± 15	58 ± 15	0.203
Male gender (N, %)	17 (56.7%)	54 (70.1%)	0.137
Ischemic aetiology (N, %)	8 (26.7%)	17 (22.1%)	0.394
De novo HF (N, %)	11 (36.7%)	57 (74.0%)	<0.001
Hypertension (N, %)	13 (43.3%)	38 (49.4%)	0.576
Diabetes (N, %)	12 (40.0%)	30 (39.0%)	0.921
BMI (kg/m^2^)	22.7 ± 2.9	24.7 ± 3.8	0.008
**Echocardiographic parameters**			
LVEF (%)	30.3 ± 15.1	28.4 ± 14.0	0.548
LVMI (g/m2)	148.2 ± 54.1	145.8 ± 35.3	0.796
LAVI (mL/m2)	63.2 ± 33.4	55.3 ± 20.7	0.143
E/E’	23.3 ± 13.0	24.5 ± 11.6	0.687
**Laboratory parameters**			
WBC (×10^3^/μL)	7182 ± 3640	7526 ± 2769	0.599
Ln hsCRP (mg/L)	1.51 ± 1.87	1.55 ± 1.32	0.915
Haemoglobin (g/dL)	12.1 ± 2.7	13.7 ± 2.6	0.007
Sodium (mmol/L)	138.7 ± 3.5	139.5 ± 4.8	0.437
Cholesterol (mg/dL)	144.5 ± 39.8	155.6 ± 43.4	0.233
Albumin (g/dL)	3.8 ± 0.5	3.8 ± 0.4	0.416
eGFR (mL/min/1.73 m^2^)	65.0 ± 26.6	69.8 ± 22.5	0.370
Ln NT-proBNP (pg/mL)	8.33 ± 0.84	7.86 ± 1.10	0.049
**Markers for pro-inflammatory response**			
Ln MIG (pg/mL)	7.32 ± 1.23	6.86 ± 1.02	0.113
Ln IP-10 (pg/mL)	5.92 ± 0.52	5.80 ± 0.62	0.425
Ln CX3CL1 (pg/mL)	3.85 ± 0.15	3.83 ± 0.17	0.730
Ln MCP1 (pg/mL)	4.52 ± 0.42	4.37 ± 0.54	0.235
Ln MIP1α (pg/mL)	2.17 ± 1.04	2.19 ± 1.30	0.937
Ln MIP1β (pg/mL)	4.34 ± 1.01	4.71 ± 0.93	0.145
Ln RANTES (pg/mL)	9.04 ± 0.13	9.03 ± 0.18	0.898
Ln Granzyme B (pg/mL)	3.73 ± 0.21	3.74 ± 0.27	0.933
Ln TNFα (pg/mL)	2.27 ± 0.40	2.28 ± 0.35	0.982
**Medications at discharge**			
RAS blockers (N, %)	23 (76.7%)	64 (83.1%)	0.305
Beta-blockers (N, %)	10 (33.3%)	53 (68.8%)	0.001
Aldosterone antagonist (N, %)	22 (73.3%)	64 (83.1%)	0.190
Calcium channel blockers (N, %)	2 (6.7%)	7 (9.1%)	0.513
Digoxin (N, %)	9 (30.0%)	14 (18.2%)	0.142
**Exercise parameters**			
Peak VO_2_ (mL/kg/min)	15.2 ± 5.4	20.0 ± 5.9	<0.001
Exercise duration (sec)	306 ± 206	505 ± 213	<0.001
Aerobic threshold time (sec)	149 ± 113	309 ± 177	<0.001
VE/VCO_2_ slope	45.6 ± 10.4	37.6 ± 9.2	<0.001
Baseline heart rate (bpm)	83.4 ± 17.9	82.7 ± 14.2	0.861
Peak heart rate (bpm)	121.8 ± 26.3	138.6 ± 30.8	0.010
HRR(1 min)	6.7 ± 10.2	19.1 ± 18.2	<0.001
HRR(2 min)	25.9 ± 17.4	37.7 ± 22.1	0.010

Values are mean ± SD or number (%). CV, cardiovascular; HF, heart failure; BMI, body mass index; LVEF, left ventricular ejection fraction; LVMI, left ventricular mass index; LAVI, left ventricular volume index; WBC, white blood cell; hsCRP, high sensitive C-reactive protein; eGFR, estimated glomerular filtration rate; NT-proBNP, N-terminal of the prohormone brain natriuretic peptide; MIG, monokine-induced by gamma interferon; IP-10, interferon gamma-induced protein 10; CX3CL1, chemokine (C-X3-C motif) ligand 1; MCP1, monocyte chemotactic protein 1; MIP1α, macrophage inflammatory protein 1α; MIP1β, macrophage inflammatory protein 1β; RANTES, regulated on activation normal T cell expressed and secreted; TNF-α, tumour necrosis factor-α; RAS, renin angiotensin aldosterone system; HRR, heart rate recovery. Discrete variables were compared using the chi-squared method, and independent Student’s t-tests were applied for the continuous variables.

### Prognostic value of HRR in relation to markers for the pro-inflammatory response

**Fig 1:**
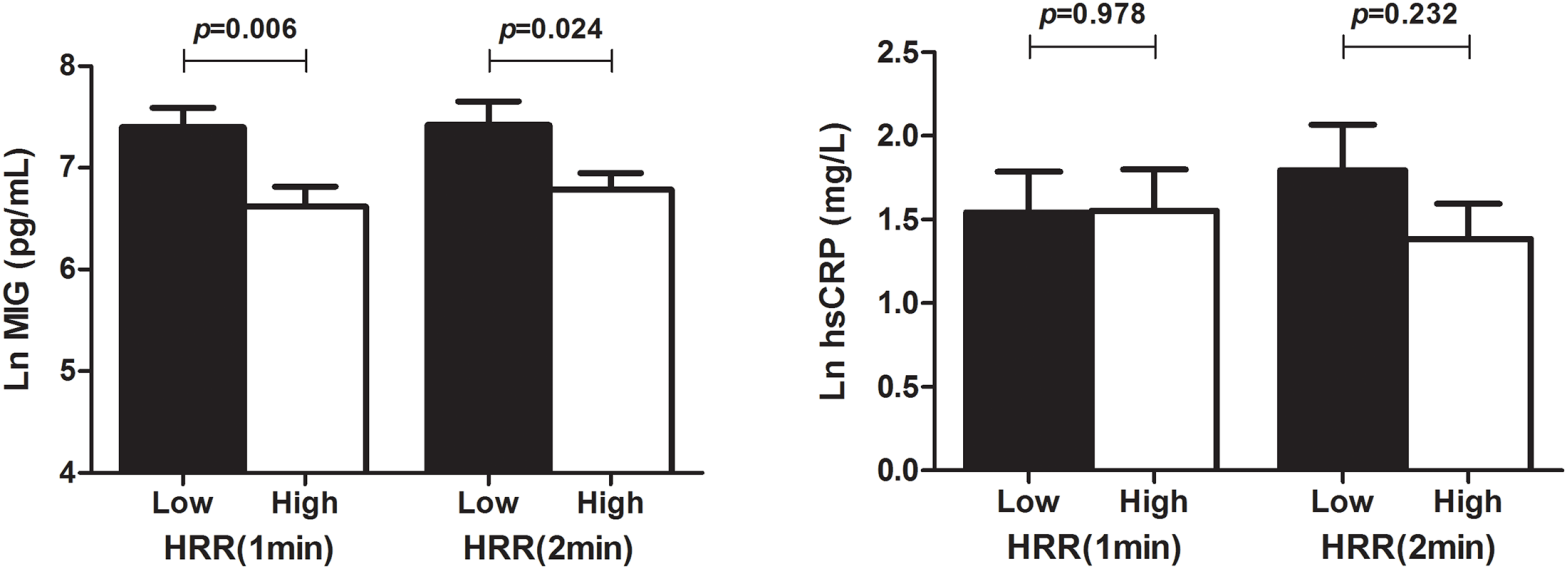
Serum levels of MIG and hsCRP according to HRR in ADHF patients.

We evaluated whether HRR is associated with the pro-inflammatory response and clinical outcomes in ADHF patients. When ADHF patients were divided by HRR based on Contal and O’Quigley's method, we found that Ln MIG, which is a T cell chemokine was significantly higher in both the low HRR(1 min) (HRR[1 min] < 13) and low HRR(2 min) groups (HRR[2 min] <27) (HRR[1 min], 7.40 ± 1.10 pg/mL vs. 6.62 ± 1.02 pg/mL, p = 0.006; HRR[2 min], 7.42 ± 1.14 pg/mL vs. 6.78 ± 1.03 pg/mL, p = 0.024). Other pro-inflammatory markers showed no significant differences relative to HRR group. Ln hsCRP, which is another marker of systemic inflammation, displayed no significant differences in the HRR(1 min) and HRR(2 min) groups (HRR[1 min], 1.54 ± 1.56 mg/L vs. 1.55 ± 1.51 mg/L, p = 0.978; HRR[2 min], 1.80 ± 1.52 mg/L vs. 1.38 ± 1.48 mg/L, p = 0.232). Serum levels of MIG and hsCRP according to HRR in ADHF patients are shown in Fig 1.

**Fig 2:**
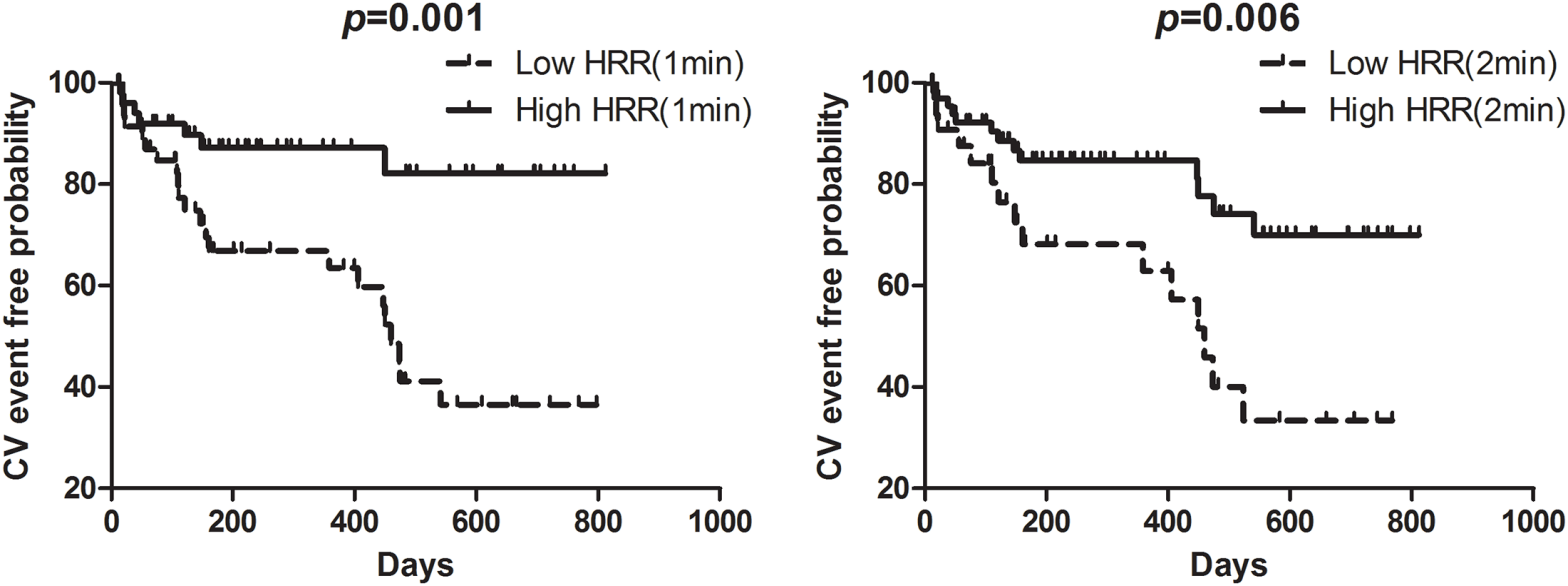
Cumulative Kaplan-Meier estimates of CV events according to HRR in ADHF patients.

Regarding the prognostic value of HRR, both low HRR(1 min) (< 13) and HRR(2 min) (< 27), were shown to be associated with poor clinical outcome (p = 0.001 for HRR[1 min], p = 0.006 for HRR[2 min]) in a Kaplan-Meier analysis (Fig 2).

To determine which factors are relevant to the clinical outcome of ADHF patients, we studied univariate and multivariate analysis for CV events. Due to the small number of CV events, we performed both enter method and stepwise method multivariate Cox regression analysis in this study (Table 2).

**Table 2:** Univariate and multivariate Cox regression analysis for CV events in patients with ADHF.

CV events	Univariate		Enter method multivariate				Stepwise method multivariate			
			with HRR(1min)		with HRR(2min)		with HRR(1min)		with HRR(2min)	
	HR (95% CI)	P-value	HR (95% CI)	P-value	HR (95% CI)	P-value	HR (95% CI)	P-value	HR (95% CI)	P-value
**Age**	1.017 (0.992–1.043)	0.178	0.998 (0.967–1.029)	0.882	0.986 (0.957–1.015)	0.327	1.009 (0.982–1.036)	0.527	0.997 (0.971–1.023)	0.800
**De novo HF**	0.255 (0.121–0.537)	<0.001	0.346 (0.131–0.916)	0.033	0.360 (0.145–0.890)	0.027	0.303 (0.138–0.666)	0.003	0.317 (0.147–0.686)	0.004
**BMI**	0.828 (0.728–0.943)	0.004	0.879 (0.749–1.031)	0.114	0.857 (0.724–1.014)	0.072	0.867 (0.760–0.988)	0.033	0.827 (0.718–0.952)	0.008
**Hemoglobin**	0.801 (0.693–0.924)	0.002	1.008 (0.799–1.272)	0.947	0.951 (0.766–1.182)	0.652				
**Ln NT-proBNP**	1.535 (1.041–2.262)	0.030	1.108 (0.691–1.779)	0.670	1.027 (0.658–1.603)	0.906				
**Beta-blocker use**	0.258 (0.120–0.555)	0.001	0.581 (0.180–1.873)	0.363	0.582 (0.199–1.700)	0.322				
**HRR(1 min)**	**0.273(0.117–0.639)**	**0.003**	**0.354 (0.136–0.921)**	**0.033**			**0.354 (0.149–0.839)**	**0.018**		
**HRR(2 min)**	**0.384(0.186–0.791)**	**0.009**			**0.400 (0.167–0.957)**	**0.039**			**0.396 (0.180–0.869)**	**0.021**

CV, cardiovascular; HR, hazard ratio; CI, confidence interval; HF, heart failure; BMI, body mass index; NT-proBNP, N-terminal of the prohormone Brain Natriuretic Peptide; HRR, heart rate recovery.

In enter method multivariate Cox regression analysis, both HRR(1min) (p = 0.033) and HRR(2min) (p = 0.039) showed independent prognostic value when controlled for age, types of heart failure, BMI, hemoglobin, NT-proBNP, and beta-blocker use. Stepwise method multivariate Cox regression analysis also revealed independent association of both HRR(1min) (p = 0.018) and HRR(2min) (p = 0.021) with the clinical outcome even after adjusting the most relevant minimum variables (age, types of heart failure and BMI) to avoid the over-fitting.

**Fig 3:**
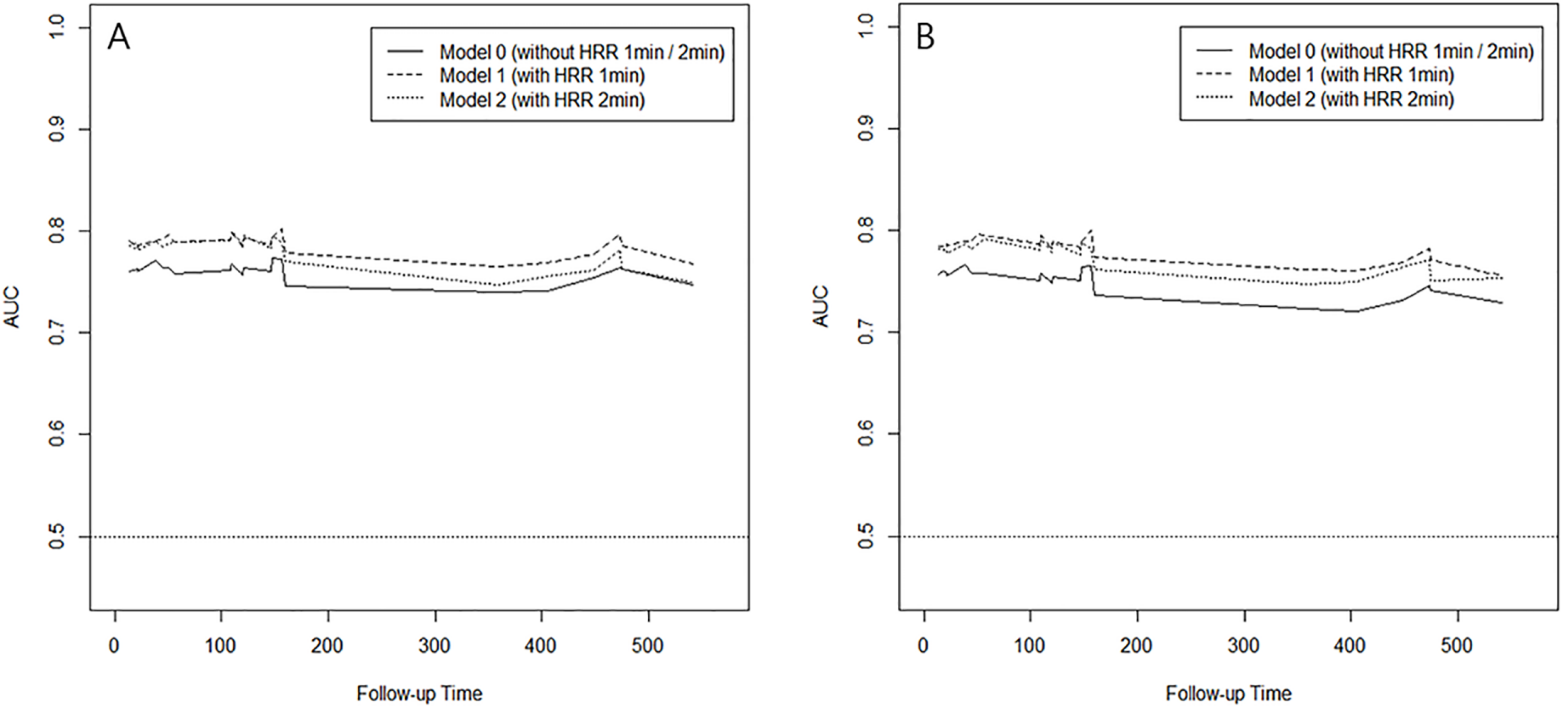
Time-dependent receiver operating curve analysis derived from the Cox regression models with and without HRR(1 min) and HRR(2 min) in enter method (A) and in stepwise method (B). The area under the curve (AUC) indicated predictive accuracy at the indicated time. Throughout the study period, the model with HRR was superior to the model without HRR in distinguishing patients who will exhibit CV events from those who will not.

In the likelihood ratio test, the full model with HRR(1 min) or HRR(2 min) exhibited significantly lower –2 log likelihood than the reduced model without HRR in both enter method (HRR[1 min], 180.827 vs. 195.486, p<0.001; HRR[2 min], 191.154 vs. 195.486, p = 0.037) and stepwise method (HRR[1 min], 209.009 vs. 225.768, p<0.001; HRR[2 min], 220.303 vs. 225.768, p = 0.019). Time-dependent ROC curves also demonstrated that the concordance probability (iAUC) increased from 0.756 (reduced model without HRR) to 0.787 (full model with HRR[1 min]) or 0.778 (full model with HRR[2 min]) in enter method and from 0.746 (reduced model without HRR) to 0.785 (full model with HRR[1 min]) or 0.774(full model with HRR[2 min]) in stepwise method. Visual inspection revealed that the full model had a higher iAUC throughout the entire follow-up period than the reduced model in both enter and stepwise method (Fig 3).

To calculate the difference in iAUC between the full and reduced models, bootstrap resampling tests were performed 1,000 times. The difference (iAUC of full model—iAUC of reduced model) was statistically significant both for HRR(1 min) (estimated difference was 0.030, 95% CI is 0.001–0.086) and HRR(2 min) (estimated difference was 0.021, 95% CI is 0.001–0.066) in enter method and also significant both for HRR(1 min) (estimated difference was 0.039, 95% CI is 0.001–0.107) and HRR(2 min) (estimated difference was 0.028, 95% CI is 0.001–0.091) in stepwise method. These findings suggest that the addition of both HRR(1 min) and HRR(2min) to the model increases the predictive accuracy for CV events.

## Discussion

The results of this study demonstrate that post-exercise HRR, which is an index of parasympathetic function, is associated with increased MIG level and independently predicts clinical outcome in prospectively and consecutively enrolled, recovered ADHF patients even after adjustment for age, types of heart failure, BMI, hemoglobin, NT-proBNP, and beta-blocker use. Moreover, the addition of HRR to a model significantly increased the predictability for CV events across the entire follow-up period.

The significant association between impaired HRR and adverse clinical outcome has been consistently reported in subjects referred for exercise testing regardless of cardiovascular disease history [3–5] and in patients with chronic heart failure [6–11]. However, the potential for referral bias exists because most of the cohorts investigated in previous studies were selected from an exercise test database. Therefore, we evaluated its prognostic value in prospectively and consecutively enrolled, recovered ADHF patients during a specific study period. Moreover, we aimed to determine the underlying mechanism that leads to a poor clinical outcome in patients with impaired HRR in terms of an exaggerated pro-inflammatory response.

The pathophysiology of how impaired autonomic dysfunction in heart failure contributes to poor clinical outcome remains poorly understood. One contributing factor could be that parasympathetic dysfunction, which is indicated by impaired HRR, might be related with excessive pro-inflammatory status. The discovery of a pathway in which cholinergic neurons inhibit exaggerated inflammation has expanded our understanding of how nervous system modulates immune responses. The nervous system reflexively regulates the inflammatory response in real time, just as it controls the heart rate and other vital functions. Immunity is coordinated by neural circuits that operate reflexively. This well-established neural circuit terminates excessive pro-inflammatory cytokine responses, preventing immune-mediated damage [12,13]. Therefore, decreased parasympathetic activity may result in exaggerated pro-inflammatory responses and increased morbidity and mortality [14–16]. However, there is a possibility that higher inflammation levels may result in impaired parasympathetic function as well.

Various markers for the pro-inflammatory response, including serum chemokine markers, granzyme B, and TNF-α, as well as hsCRP, were evaluated in this study. Among them, MIG, which is one of the C-X-C chemokine receptor type 3 (CXCR3)-binding T cell chemokines, showed significant differences across the HRR groups unlike hsCRP and TNF-α. Recent human studies reported that circulating MIG levels are increased in newly diagnosed, treatment-naïve hypertensive patients [18], and are independently associated with the coronary artery calcium score [19] or carotid intima media thickness [20]. Recently, the pathogenic role of CD4^+^ T cells in pressure overload-induced cardiac remodelling and in the transition to heart failure was demonstrated in animal models [21]. Moreover, the enhanced CD4^+^ T cell activation observed in patients with heart failure in proportion to severity suggests that T cell-driven inflammation is one important pathophysiology of heart failure [22,23]. Consequently, our findings that patients with autonomic dysfunction showed increased serum MIG levels further support the involvement of T cell-driven inflammation in patients with heart failure.

It has been suggested that specific medication, such as beta-blockers, may influence the HRR. Lipinski, et al. [7] and Kubrychtova, et al. [10] showed improved HRR in patients taking beta-blockers while Racine, et al. [24] reported that beta-blocker therapy does not significantly improve HRR, suggesting that controlling for beta-blocker use in the evaluation of HRR may not be necessary. In our study, both HRR(1 min) and HRR(2 min) showed no significant differences between beta-blocker users and non-users. However, impaired post-exercise HRR independently predicted clinical outcome irrespective of beta-blocker use in this study.

Several device-based therapies to target specific aspects of autonomic imbalance are actively under investigation in patients with heart failure. Among them, vagal nerve stimulation has been shown to improve clinical heart failure symptoms, New York Heart Association (NYHA) heart failure class, and evidence of left ventricular reverse remodelling in an open label pilot study [25]. More recently, the results of two other human vagal nerve stimulation studies, NECTAR-HF [26] and ANTHEM-HF [27], were reported. While NECTAR-HF did not show improvements in the primary endpoint of reduction in left ventricular end-systolic diameter (LVESD), ANTHEM-HF demonstrated significant improvement in left ventricular ejection fraction and LVESD in left and right vagal stimulation of chronic symptomatic heart failure patients. Currently, the INOVATE HF clinical trial [28], using the CardioFit System, is being conducted in 90 centres worldwide to evaluate its long-term safety and efficacy in patients. This trial will provide more definitive data on the safety and efficacy of vagal nerve stimulation in patients with heart failure. Another recent first-in-human trial of high thoracic spinal cord stimulation revealed that this approach is safe and can potentially improve symptoms, functional capacity, and cardiac function in patients with advanced HF [29]. Regarding the use of device-based therapies to modulate autonomic dysfunction, it is important to investigate the proper patient population, one that will benefit from these device therapies. Post-exercise HRR, which is an index of parasympathetic function, can be used in patient selection for autonomic modulation similar to left bundle branch block pattern QRS morphology and QRS duration for cardiac resynchronization therapy.

The present study has several potential limitations. First, due to the maximal exercise protocol, the intensity of exercise by each patient is not controlled. Second, our study population cannot represent all heart failure patients. The analysed subjects were hospitalized and ambulatory, recovered ADHF patients. Moreover, these data were collected from a Korean population. The generalizability of the current findings to other populations (e.g. Caucasian populations) is unknown. Third, the lack of adjustment for diet pattern and socioeconomic status might be noted as well. Fourth, due to the small number of CV events, our multivariate Cox regression model included a limited number of variables. Peak VO_2_, which is a well-known prognostic factor in patients with HF is not adjusted in the multivariate analysis due to the significant co-linearity with both HRR(1 min) and HRR(2 min). Finally, lack of other parasympathetic activity measurements, such as heart rate variability or baroreflex sensitivity, could be another limitation of this study. However, we evaluated the prognostic value of post-exercise HRR in prospectively and consecutively enrolled, recovered ADHF patients, thereby minimizing the potential for referral bias. Moreover, we sought to examine the underlying mechanism that leads to poor clinical outcome in patients with impaired HRR in terms of ‘cholinergic anti-inflammatory pathway’.

## Conclusions

Post-exercise HRR is an index of parasympathetic function associated with clinical outcome in patients with chronic heart failure. However, its prognostic value has not been confirmed in prospectively and consecutively enrolled ADHF patients. Here, we confirm that impaired post-exercise HRR is associated with an exaggerated pro-inflammatory response and independently predicts clinical outcome in patients with ADHF. Measurement of post-exercise HRR is a simple and feasible assessment, which might have important implications on risk stratification of ADHF patients.
